# Global Geometric Morphometric Analyses of the Human Pelvis Reveal Substantial Neutral Population History Effects, Even across Sexes

**DOI:** 10.1371/journal.pone.0055909

**Published:** 2013-02-07

**Authors:** Lia Betti, Noreen von Cramon-Taubadel, Andrea Manica, Stephen J. Lycett

**Affiliations:** 1 Department of Anthropology, School of Anthropology and Conservation, University of Kent, Canterbury, United Kingdom; 2 Department of Archaeology and Anthropology, University of Cambridge, Cambridge, United Kingdom; 3 Department of Zoology, University of Cambridge, Cambridge, United Kingdom; State University of New York College at Oneonta, United States of America

## Abstract

Recent applications of population genetic models to human craniodental traits have revealed a strong neutral component to patterns of global variation. However, little work has been undertaken to determine whether neutral processes might also be influencing the postcranium, perhaps due to substantial evidence for selection and plastic environmental responses in these regions. Recent work has provided evidence for neutral effects in the pelvis, but has been limited in regard to shape data (small numbers of linear measurements) and restricted only to males. Here, we use geometric morphometric methods to examine population variation in the human os coxae (pelvic bone) in both males and females. Neutrality is examined via apportionment of variance patterns and fit to an Out-of-Africa serial founder effect model, which is known to structure neutral genetic patterns. Moreover, we compare males and females directly, and the true versus false pelvis, in order to examine potential obstetrical effects. Our results indicate evidence for substantial neutral population history effects on pelvic shape variation. They also reveal evidence for the effect of obstetrical constraints, but these affect males and females to equivalent extents. Our results do not deny an important role for selection in regard to specific aspects of human pelvic variation, especially in terms of features associated with body size and proportions. However, our analyses demonstrate that at a global level, the shape of the os coxae reveals substantial evidence for neutral variation. Our analyses thus indicate that population variation in the human pelvis might be used to address important questions concerning population history, just as the human cranium has done.

## Introduction

In recent years, the study of human morphological variation has seen a shift of attention from mainly selective processes to the neutral component of shape variation (i.e., due to genetic drift and migration) [1]. Most of this work has been carried out on craniodental traits, showing how variation in cranial and dental morphology largely mirrors neutral genotypic variation in modern human populations (e.g., [2]–[14]). Neutrality of cranial variation at a global level has been inferred on the basis of three main approaches [15]: examination of the apportionment of phenotypic variance at different geographic levels, directly comparing phenotypic distance to neutral genetic distance, and testing for the preservation of serial founder events which occurred during the expansion of our species out of Africa.

Lewontin [16] was the first to describe the apportionment of genetic variance within human populations and between major geographic regions. Specifically, he showed how the large majority of human genetic diversity is present at the local population level (∼85%), while only about 6% of variation separates what had conventionally been conceived of as the major “racial” groups [16]. The same method has since been applied to a range of neutral genetic markers (e.g., [17], [18]) and to craniometric variation, confirming the same general apportionment pattern [2], [3], [7]. Conversely, it has been shown that traits under selection, for example human skin color variation, tend to have relatively small within-population diversity (∼9%), while most of the variation lies between populations and between geographic regions [3]. Therefore, despite the fact that the microevolutionary significance of Lewontin’s findings may have been overstated (e.g., [19]–[21]), variance apportionment patterns do distinguish effectively between largely neutrally evolving phenotypes, such as human cranial shape, and strongly selected phenotypes, such as human skin color.

Direct comparison of genetic and phenotypic distances between populations also confirmed a substantial neutral signal in cranial variation [6], [8], [9], [13], [14], [22], [23]. Moreover, cranial differences between human populations were shown to follow the same Isolation By Distance (IBD) pattern previously described for genetic distance [24], [25], whereby a decrease of genetic and phenotypic similarity between pairs of populations accompanies increasing geographic separation, due to reduced gene flow over larger distances [4], [26].

Finally, within-population cranial diversity, like genetic diversity, reveals a clear signature of the expansion of the human species out of the African continent. Genetic diversity (heterozygosity) was shown to decrease with increasing distance from sub-Saharan Africa, a pattern that has been explained as the signature of serial founder events that accompanied the colonization of other continents [27]–[29]. Subsamples of established populations would move to new areas, founding new communities that would in time be the origin of further expansion by a new sub-population. With each founding event, some genetic diversity is lost at random, creating the described worldwide pattern. The presence of the same, if somewhat weaker, signature in craniodental variation is additional evidence of the overall neutrality of cranial variation [10]–[12], [30].

Only very recently has the same interest in neutral processes shaping human variation been extended to the postcranium [31], where variation in different populations has otherwise been addressed mainly in terms of selection and plastic reactions to the environment (e.g., [32]–[38]). Betti et al. [31] showed how various regions of the postcranium, specifically the pelvis and long bones, reflect differently the signature of the Out-of-Africa (OoA) expansion. In particular, the pelvis, like the cranium, reveals a clear signature of the past population expansion, while the pattern is absent in long bones, which in turn seem to be affected by climate. The preservation of the signature of ancient demographic events in the pelvis, as for the cranium, suggests a substantial fit to a neutral model of phenotypic variation in this anatomical region.

The shape of the pelvis has long been the focus of attention of paleoanthropologists (e.g. [39]–[49]), as well as physical anthropologists interested in modern human biology and variation (e.g., [33], [50]–[56]). Being involved in two fundamental activities, locomotion and childbirth, the pelvis is under complex and conflicting constraints, often referred to as the obstetrical dilemma [57]–[59]. While bipedal locomotion privileges narrow pelves, parturition of a large-brained neonate requires that the birth canal be wide enough to allow the passage of the skull. These conflicting factors led to a close fit between the size of the modern human birth canal and the neonate skull which is absent in other apes, accompanied by increased danger of complications during childbirth. This problem may also additionally be confounded by climatic adaptation in humans [48]. Indeed, there is evidence that climate independently affected the form of the pelvis following the colonization of high latitude regions by modern humans. Various studies have found that human populations living in colder climates have relatively wider pelves that help reduce heat loss by decreasing the surface-to-volume ratio of the body [32], [33], [35], [48], [60]. Acetabular diameter is also related to body mass [61], [62] and, therefore, could also be subject to climatic adaptation via Bergmann’s [63] rule.

Despite the large body of literature suggesting that selection and evolutionary constraints have affected the morphology of the human pelvis, the results of Betti et al. [31] clearly point to the persistence of a neutral signal, related to population history, in pelvic shape variation that has not been completely erased by selection. However, such results were obtained with a less than ideal dataset, with strong limitations in respect to shape representation (only three pelvic measurements) and in the absence of female individuals for a comparison between the sexes. Hence, further and more detailed testing of the neutral model is warranted.

In this study, in the absence of matched genetic samples for our phenotypic data, the fit to a neutral model for global shape variation in the human pelvis will be assessed via two of the aforementioned methods. The presence of the Out-of-Africa (OoA) demographic signature on the pelvis will be examined in greater detail and in both sexes, on a dataset compiled to maximize shape definition (using 3D morphometric data of the os coxae) and to better represent global variation (larger and a more homogeneous distribution of population samples). These data also facilitate a methodological comparison of 3D geometric (landmark) data versus inter-landmark distance data in addressing questions of this type. Additionally, an overview of the apportionment of variance will also be carried out, in order to evaluate whether global patterns of within- and between-population pelvic shape variation apportion in a similar way to neutrally evolving genotypes/phenotypes or to non-neutral phenotypes such as human skin color variation.

**Figure 1:**
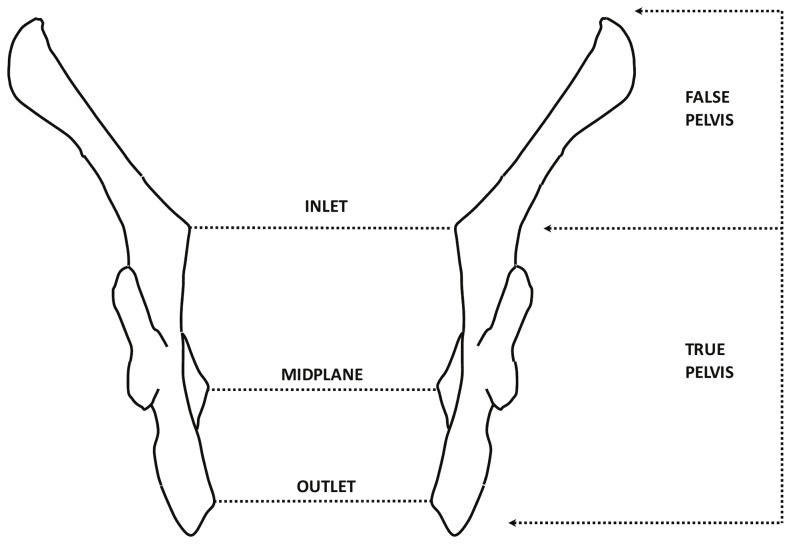
Diagram of the true and false pelvis. Inlet, midplane and outlet refer to the different planes of the birth canal (figure redrawn after [51], [65], [66]).

In addition, we also consider the possible confounding effect of obstetrical constraints on neutral variation. Obstetrical constraints may have a direct effect on internal population variance due to strong stabilizing selection [64], whose effect is to reduce genetic and phenotypic variance. For instance, Kurki [65] has shown that in populations characterized by small body size, females retain similar dimensions of the birth canal as in larger-bodied populations. This suggests that some obstetrical dimensions are evolutionary protected and preserved, thus helping maintain viable canal dimensions in small-size populations. Indeed, complications during parturition are expected to have a considerable direct effect on the fitness of a woman, which would be decreased *in primis* by her death, but also by the death or parturition-induced disabilities of the newborn [64], [65]. Given the potential strength of the selective pressure acting on the pelvis, we can expect that obstetrical constraints might affect the shape of the os coxae, reducing within-population variability, at least in females. Obstetrical constraints may be expected to act mainly on the area of the birth canal, and in general on what is often referred to as the “true pelvis” (e.g., [66]). The cavity of the pelvis is in fact often divided into two areas: the “true pelvis” (or “lesser pelvis”), which includes the space internal to the pelvic girdle situated below the pelvic brim, and the “false pelvis” (or “greater pelvis”), which includes the internal pelvic space above the pelvic brim (Figure 1). The part of the os coxae that contributes to the true pelvis might, therefore, be under stronger constraints than the part not directly involved in parturition (false pelvis). The presence of such constraints could, therefore, decrease the strength of the neutral pattern in the female phenotype, particularly in the true pelvis. We test these predictions here by comparing male and female pelvic shape in terms of relative goodness of fit to the serial founder effect (OoA) model, both in terms of overall pelvic shape, and in terms of two alternative landmark configurations that deliberately differ in respect to coverage of the true pelvis versus the false pelvis.

## Materials and Methods

Morphometric data for the os coxae were collected by one of us (LB) in 1,443 adult individuals, 891 males and 552 females, curated at several osteological collections (Table 1). Only individuals whose iliac crest was completely fused were measured in this study. The dataset comprises 27 male and 20 female globally distributed population samples, with a minimum sample size of 16 individuals per population, and an average sample size of 33 and 27.6 individuals for males and females, respectively.

**Table 1:** Population samples and institutions where material is hosted.

Region	Males	Females	Institution
*Africa*			
Botswana, Tswana	33	30	UW
Egypt dynastic	34	17	AMNH, NM
Kenya, Kykuyu	40	30	NMK
Lesotho, Sotho		34	UW
Malawi	33		UW
Nubia	33	25	NM
South Africa, Khoi-San	26	21	AMNH, MGM, NHM,UW
South Africa, Venda	29		UW
Swaziland	39	20	UW
*Europe*			
Austria	70	16	CMNH, NM
France	28	23	MdH
Ireland	28		CMNH
Italy	33		CMNH, MNdAE
Portugal	42	42	CU
Western Russia	26		CMNH
*Asia*			
Ainu, Japan	23	20	KU, TU
India	28		AMNH, MdH, MoM,NHM, UW
Iran	32	22	UP
Japan	45	37	AMNH, KU, MdH,NH
Thailand	37	36	CMU, MdH
*America*			
Alaska, Point Hope	38	35	AMNH
Argentina, Patagonia	33	23	MdH, MNdAE
Canada, Sadiermiut	24	24	MCC
Chile, Fuegians	17		MNdAE, NHM, UR
Native Californians	36	31	UCB
Peru	31	33	MdH, UCB
South Dakota, Arikara	35	33	UTK
Tennessee, Late Mississippian	18		UTK

AMNH = American Museum of Natural History, New York; CMNH = Cleveland Museum of Natural History, Ohio; CMU = Chiang Mai University, Thailand; CU = Coimbra University, Portugal; KU = Kyoto University, Japan; MCC = Musée Canadien des Civilisations, Gatineau, Canada; MdH = Musée de l’Homme, Paris, France; MGM = McGregor Museum, Kimberley, South Africa; MNdAE = Museo Nazionale di Antropologia e Etnologia, Firenze, Italy; MoM = San Diego Museum of Man, California; NHM = Natural History Museum, London, UK; NM = Naturhistorishes Museum, Wien, Austria; NMK = National Museum of Kenya, Nairobi, Kenya; TU = Tokyo University, Japan; UTK = University of Tennessee at Knoxville; UCB = University of California at Berkeley; UP = University of Pennsylvania at Philadelphia; UR = University of Rome “La Sapienza”, Italy; UW = University of Witwatersrand, Johannesburg, South Africa.

**Figure 2:**
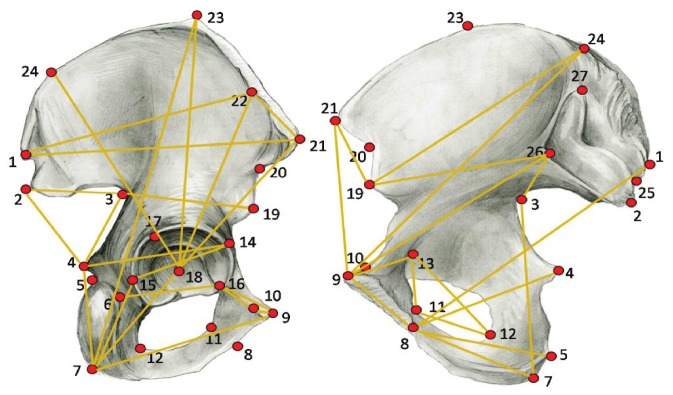
Landmarks and inter-landmark distances. Landmarks and linear distances used in the study are shown on right os coxae (lateral view shown on left, medial view shown on right).

Sex of the individuals, whenever unknown, was assessed visually by LB following standard non-metric methods [67]–[69]. The morphometric data consist of a set of 27 landmarks on the os coxae (Figure 2, Table 2), obtained using an Immersion 3D Microscribe digitizer. The data were collected unilaterally, for the best preserved side (i.e. complete), allowing inclusion of individuals with an incomplete preservation of the entire pelvic girdle. The set of landmarks was specifically designed to represent the complex shape of the os coxae and minimize inter-observer error. Five repeat measurements of all landmarks were performed on three os coxae from different individuals, and landmarks associated with a standard deviation over 1 mm were excluded [70]. Differences in position, rotation and size between individual configurations were corrected by generalized Procrustes superimposition [71]–[74] in Morphologika 2.5 [75], in males and females separately. The centroid size for each os coxae configuration was retrieved after the superimposition. New morphometric variables in the form of Principal Component (PC) scores were extracted from the whole 3D configuration of the os coxae by performing a Principal Component Analysis (PCA) in tangent shape space on the covariance matrix of the Procrustes coordinates. The obtained PC scores, representing 100% of the total shape variance, were used as morphometric data in the following analyses.

**Table 2:** Definition of landmarks used in this study.

Landmark	Definition
1	Apex of the posterior superior illiac spine*
2	Apex of the posterior inferior illiac spine*
3	Point of maximum curvature in the greater sciatic notch*
4	Tip of the ischial spine. The lower point of the tip is taken when the spine is not triangular in shape*
5	Point where the transverse ridge meets the medial edge of the ischial tuberosity*
6	Apex of the ischium tubercle
7	Farthest point of ischial curve from the centre of the obturator foramen
8	Most inferior point on the inferior edge of the medial aspect of the pubic symphysis*
9	Most superior point on the superior edge of the medial aspect of the pubic symphysis*
10	Apex of the pubic spine
11	Most anterior point of the obturator foramen
12	Most posterior point of the obturator foramen
13	Most superior point of the obturator foramen
14	Point on the acetabulum margin corresponding to where ilium and ilio-pubic ramus meet
15	Point on the acetabulum margin furthest away from landmark 14
16	Most inferior point of the anterior end of the lunate surface of the acetabulum
17	Point on the acetabulum margin furthest away from landmark 16
18	Center of the acetabular fossa
19	Apex of the anterior inferior illiac spine*
20	Deepest point of the interspinal notch
21	Apex of the anterior superior illiac spine*
22	Midpoint of the supero-lateral edge of the cristal tubercle*
23	Most superior point of the illiac crest, in measuring position
24	Point where the lateral margin of the iliac crest meets the superior end of the posterior gluteal line*
25	Point where the posterior margin of the auricular surface meets the margin of the ilum.
26	Point where the arcuate line meets the auricular surface of the ilium*
27	Most postero-superior point on the auricular surface of the ilium*

The os coxae is orientated in lateral view and the posterior iliac spines together with the edge of the ischial tuberosity lie on an imaginary y-axis with respect to the observer. When possible, Weaver’s [60] landmark definitions have been used (highlighted by an asterisk, [60]).

### Accuracy of Visual Sex Determination

Visual methods of sex assessment were developed originally mainly for individuals of European ancestry, and their efficacy in individuals from different continents is largely unknown. In order to test for the accuracy of visual determination of sex in the global dataset under study, a specific test was developed using the available 3D data. Male and female individuals were pooled together and subjected to full Procrustes superimposition. Discriminant analysis was run in R version 2.14 [76] on the PC scores of a subset of the individuals (169 males and 165 females) of known sex obtained from forensic or cemetery collections (Table 3, DB1). The selected individuals were chosen from populations of different continents, in order to represent ethnic and geographic variation. The resulting discriminant function was applied separately to the remaining individuals of known sex available in the dataset (Table 3, DB2), and to all individuals whose sex had been visually estimated (Table 3, DB3). The results of the discriminant function were compared to the actual sex of one dataset and the estimated sex of the other, and the rate of individuals with matching sex was calculated. If visual sex determination has a high accuracy, we can expect the discriminant function to give a similar rate of matching sex individual in both datasets. The proportion of individuals with the correct matching sex was very similar in the known and unknown sex database (99.04% and 98.87% respectively), supporting the validity of the visual sex determination.

**Table 3:** Subsets of the original dataset used to test the accuracy of visual sex determination.

Population samples	Males (N)	Females (N)	Total (N)
DB1	169	165	334
*Africa*	*62*	*64*	
*Europe*	*70*	*65*	
*Asia*	*37*	*36*	
DB2	253	59	312
*Africa*	*79*	*21*	
*Europe*	*157*	*10*	
*Asia*	*17*	*28*	
DB3	469	328	797
*Africa*	*126*	*92*	
*Europe*		*6*	
*Asia*	*111*	*51*	
*Americas*	*232*	*179*	

The numbers refer to the number of individuals (N). DB1: known-sex individuals used to derive the discriminant function; DB2: different subset of known-sex individuals; DB3: estimated-sex individuals.

### Apportionment of Variance

Population samples (Table 1) were assigned to different continents (Africa, Europe, Asia, and America) and the relative apportionment of variance at the population and continent level was calculated as in [3], [77].

The method is based on a quantitative genetics model, where each locus has an equal and additive effect. It requires the computation of an R matrix of scaled variances and covariances around the regional mean allele frequencies [78], [79]. In the absence of an estimate of the additive genotypic covariance matrix, the phenotypic covariance matrix is used, assuming total heritability. Given the small sample size of some of the populations, a correction for sampling error was included in the calculation of the R matrix, as suggested by [77]. Unbiased F_ST_ values were extracted from the R matrix as the weighted average diagonal of the R matrix:
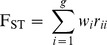



Where *g* is the number of populations, and *w* is the relative population size of population *i*. In the absence of any specific information, identical *population* size was assumed for all populations and *w* was set as a vector of 1. The apportionment of variance was explored at a hierarchical level, following [16] and subsequent analogous works:

AR = variance among geographic regions (in this case continents);

AL = variance among local populations within regions;

WL = variance within local populations.

If F_RT_ is F_ST_ calculated among regions, and F_LT_ is F_ST_ calculated among local populations, then:

AR = F_RT_

AL = F_LT_–F_RT_

WL = 1–F_LT_

### Assessing Fit to the Out-of-Africa Model

Within-population phenotypic diversity was calculated as the trace of the variance-covariance matrix of the PC scores [80], [81], which is effectively a sum of each PC’s within-population variance. Following [10], all potential origins of human expansions were represented as a grid of equally spaced points (5° latitude/longitude) distributed over Africa, Eurasia, Australia and the Americas. Geographic distance from each potential origin was calculated as the shortest route over land avoiding areas with an altitude greater than 2,000 m (computed using the graph theory approach described by [82]).

To test for the signature of the serial founder effect, a linear regression analysis was implemented in R, where phenotypic multivariate variance was dependent on geographic distance from the origin of expansion. The analyses were repeated for all possible origins, in order to identify the origin associated with the lowest AIC (Akaike Information Criterion, [83]) for both males and females.

Analogous published works on the cranium used linear measurement data to quantify shape variation [10], [12], [30]. To allow a more direct comparison with previous studies, 39 linear Euclidean distances between landmarks were extracted from the 3D data (Figure 2). The measurements were size adjusted by dividing each inter-landmark distance by centroid size (i.e. the square root of the sum of the squared Euclidean distances from each landmark to the centroid). Following the Relethford-Blangero [79] method, the inter-landmark distances were subject to a z-score transformation which standardizes all measurements by subtracting the population’s mean and dividing the result by the population’s standard deviation, erasing the effect of scale differences and allowing a direct comparison between measurements. Within-population variance was computed for the linear measurements and regressed on the distance from the centroid of the area within 4 points of delta AIC of the single most supported origin.

### Obstetrical Constraints

**Figure 3:**
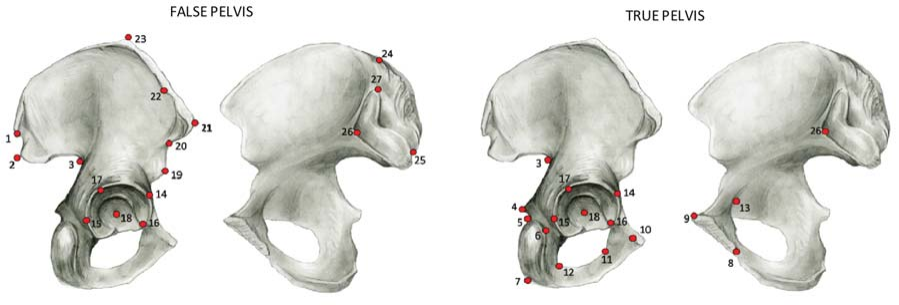
Landmark configurations for regions of the os coxae associated with the false and true pelvis. The acetabular region is included in both configurations.

To test for the possible effect of obstetrical constraints on the os coxae, two new configurations of 17 landmarks were created from the full set of landmarks, broadly related to the shape of the true and false pelvis (Figure 3). The region of the acetabulum was included in both configurations, therefore maintaining useful shape information regarding the position of the landmarks in respect to the axis of the body. The OoA model was then tested separately on both configurations, using the methods described above.

## Results

### Apportionment of Variance

The relative apportionment of within- and between-population phenotypic variance at a regional (continental) and local level is very similar for the os coxae as to the pattern described for neutral genetic markers and craniometric data, with the large majority of shape variance found within local populations (i.e., >90%; Table 4). This contrasts sharply with the apportionment pattern for skin color, where within-population variation only accounts for less than 10% of the total diversity. Therefore, these results are consistent with the suggestion that global patterns of variation in the shape of the os coxae can largely be attributed to neutral evolutionary processes.

**Table 4:** Apportionment of genetic and phenotypic variance.

			Variance components (%)		
Data	Reference	Number of regions	Among regions (AR)	Among local populations within regions (AL)	Within local populations (WL)
Blood polymorphisms	[16]	7	6.3	8.3	85.4
Blood polymorphisms	[84]	6	10.4	5.6	84.0
Microsatellite DNA	[17]	5	10.0	5.5	84.5
RFLPs, 16 loci	[17]	5	8.0	8.4	83.6
RFLPs, 79 loci	[17]	4	11.7	3.9	84.5
Craniometrics*h^2^ = 0.55	[3]	6	14.6	6.7	78.8
Skin color h^2^ = 0.66	[3]	5	87.9	3.2	8.9
Pelvic shape (males) h^2^ = 1	Present study	4	2.6	4.8	92.6
Pelvic shape (females) h^2^ = 1	Present study	4	3.3	6.4	90.3

Results as reported in some of the cited works and in the present study. Only studies comparing at least 4 main geographic regions were included. The present study assumes complete heritability thus generating minimum F_ST_ values. Craniometric average heritability based on [103] and skin color heritability based on [104].

### Out-of-Africa Model

**Figure 4:**
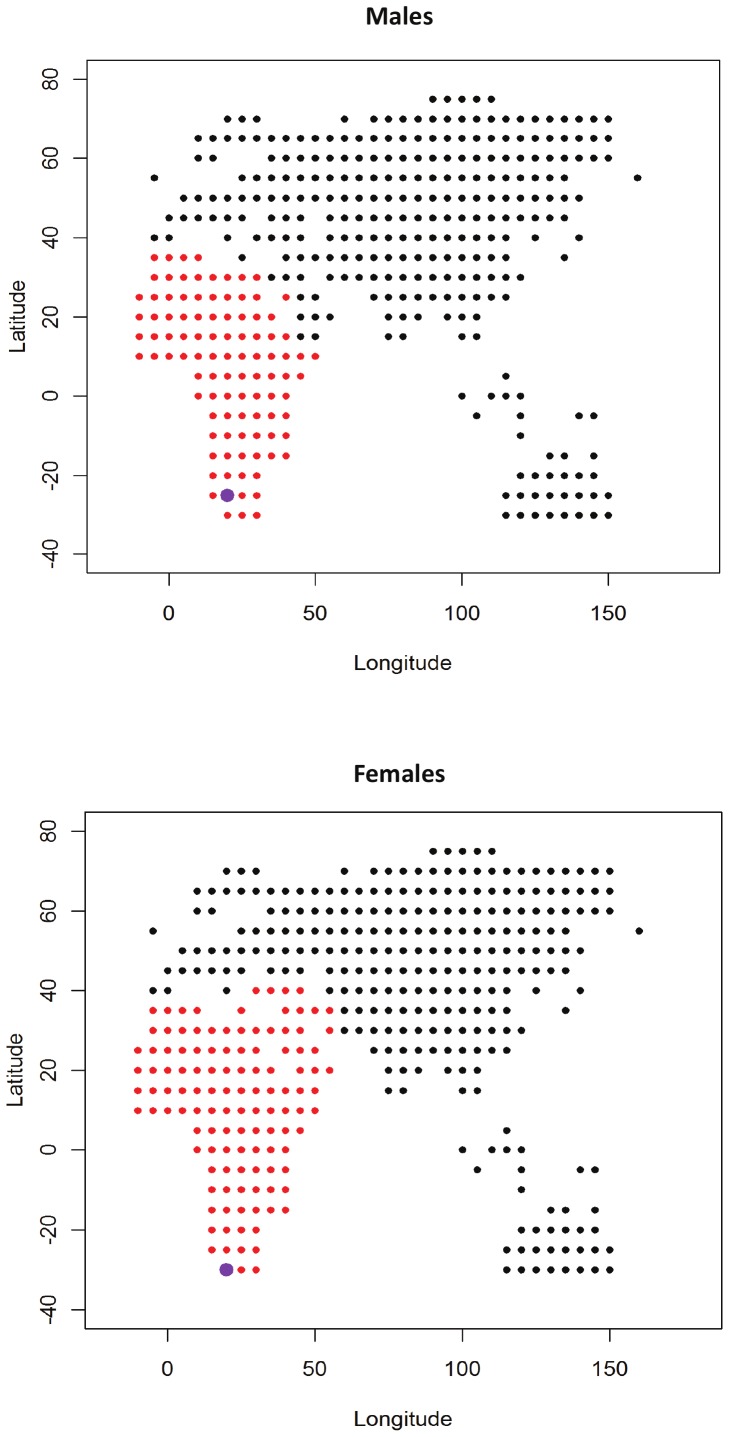
Points of origin of human expansion tested in males (above) and females (below). The origin that returned the highest correlation between phenotypic variance and geographic distance is highlighted with a larger dot. The origins that gave a regression model within 4 points of delta AIC from the best origin are highlighted by red dots. Americas not shown but included in analysis.

The test of the iterative founder effect model on all possible origins gave very similar results in males and females (Figure 4). In both sexes, the “best” origin (i.e., the origin associated with the lowest AIC) was found in South Africa (25°S 20°E for males and 30°S 20°E for females). Expanding the confidence interval to all origins within 4 points of AIC from the best model returned an area that matches very closely the whole African continent, with a centroid in central sub-Saharan Africa (5°N 25°E).

**Figure 5:**
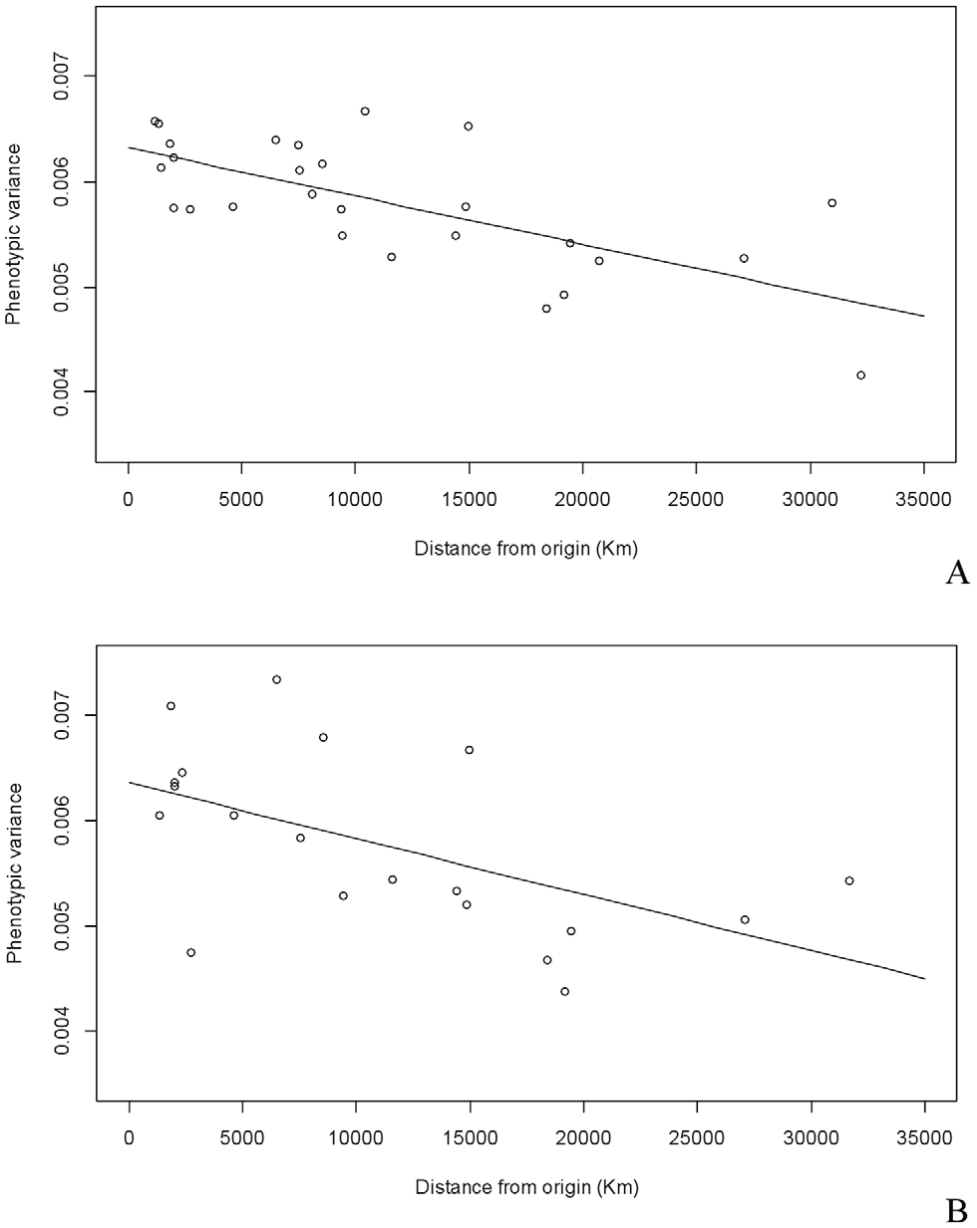
Out-of-Africa pattern. Plot of within-population phenotypic variance on distance from the sub-Saharan African centre of origin for males (A) (r^2^ = 0.466; p-value <0.001) and females (B) (r^2^ = 0.305; p-value = 0.012) (Table 5). The difference between males and females disappears when equal numbers of populations (i.e. 19) are used for males (r^2^ = 0.292; p-value = 0.017) and females (r^2^ = 0.284; p-value = 0.019) (Table 5).

Within-population variance shows a significant decline with increasing distance from the centroid of the most supported area of origin (i.e. 5°N 25°E) in both males and females (Table 5, Figure 5), confirming the results of [31] in showing a clear neutral signature of the OoA expansion in pelvic shape variation. The pattern appears stronger in males than in females (r^2^ = 0.466 and r^2^ = 0.305, respectively). Repeating the analyses on inter-landmark distances gave very similar results (r^2^ = 0.376 in males and r^2^ = 0.315 in females; Table 5).

**Table 5:** Results of linear regression models.

MALES	R^2^	P-value	FEMALES	R^2^	P-value
PhVar∼Dist	0.466	<0.001	PhVar∼Dist	0.305	0.012
PhVar∼Dist (linear)	0.376	<0.001	PhVar∼Dist (linear)	0.315	0.010
PhVar∼Dist (19 pop.)	0.292	0.017	PhVar∼Dist (19 pop.)	0.284	0.019
PhVar∼Dist (linear, 19 pop.)	0.318	0.012	PhVar∼Dist (linear, 19 pop.)	0.341	0.009
PhVar∼Dist (true pelvis)	0.382	<0.001	PhVar∼Dist (true pelvis)	0.210	0.042
PhVar∼Dist (false pelvis)	0.419	<0.001	PhVar∼Dist (false pelvis)	0.313	0.010

PhVar: phenotypic variance; Dist: distance from the African origin; linear: analysis performed on inter-landmark distances; 19 pop.: analysis performed on a subsample of 19 populations for which both male and female individuals were available.

The possible presence of a bias towards lower variances in smaller samples could in principle affect the OoA pattern. A simple regression analysis, however, showed no significant relationship between phenotypic variance and sample size (r^2^ = 0.097, p-value = 0.11 in males; r^2^ = 0.066, p-value = 0.281 in females) nor between sample size and distance from Africa (r^2^ = 0.070, p-value = 0.182 in males; r^2^ = 0.042, p-value = 0.385 in females).

The results indicate that the neutral demographic pattern is stronger in males than in females. To test if the difference could be due to the more numerous population samples available for males (N = 27) compared to females (N = 20), the analyses were repeated on a subset of 19 populations that included individuals from both sexes. No difference between the sexes was highlighted in landmark data (r^2^ = 0.292 and r^2^ = 0.284 in males and females respectively) nor the linear distances (r^2^ = 0.318 and r^2^ = 0.341 in males and females respectively).

The analyses showed a lower OoA signal in the true pelvis with respect to the false pelvis (Table 5). Moreover, average within-population variance is lower in the true pelvis (0.0026 in both males and females using 19 populations) than in the false pelvis (0.0041 and 0.0040 in males and females, respectively using 19 populations). The results are in line with what would be expected if obstetrical constraints were indeed affecting the true pelvis. However, results were virtually identical between males and females, both in the strength of the OoA signal and in within-population pelvic variation (0.0058 and 0.0057 in males and females, respectively, using 19 populations) suggesting that the constraints, if present, do not affect female pelvic shape to a greater extent than male shape.

## Discussion

It is clear from the results that at a global level, the shape of the os coxae reflects the effects of ancient demographic events on genetic diversity, as expected under a model of neutral phenotypic evolution. The pattern of variance apportionment at a regional and local level is consistent with previous analyses of neutral genetic markers and (largely neutral) craniometric traits [3], [17], [18], [84], [85], and opposite to the pattern shown by skin color, which has been attributed to strong natural selection (Table 4) [3], [86]. The analysis assessing all possible origin points found that the best supported origin lay in sub-Saharan Africa, consistent with previous assessments of neutral genetic markers [10] and also equivalent analyses of craniometric data [10], [12]. Phenotypic diversity, moreover, declines with increasing distance from sub-Saharan Africa (r^2^ = 0.31–0.47), following the same pattern described in neutral genetic markers [27]–[29] and craniodental traits [10]–[12], [30]. The results are particularly striking as the pattern appears stronger than found previously on a more limited set of pelvic measurements (r^2^ = 0.15) [31], or even in larger studies of cranial variation (r^2^ = 0.19–0.28) [12], [30]. These results would obviously benefit from further investigation via directly matched global samples of cranial and postcranial remains. However, it is worth emphasizing that given the small sample size of some groups, we can expect the presence of some “noise” in the analyses, which would primarily affect the regression coefficient of the OoA model. Therefore, the results reported in Table 5 should be considered as minimal values.

One possible reason for the difference highlighted between this study and analogous tests of the OoA pattern on the pelvis and the cranium could be the nature of the data themselves. All previous studies made use of linear measurement data collected using caliper-based traditional morphometric methods. Repeating the analyses on inter-landmark distances, however, returned very similar results (r^2^ = 0.32–0.37), again supporting a very strong OoA pattern in the pelvis. It appears, therefore, that independently of the specific category of data, global shape variation of the os coxae reflects the neutral signature of population history in *Homo sapiens*. A comparison with the weaker OoA signal found by [31] using only three pelvic measurements suggests that the number and the choice of traits, more than the type of data employed, affects the overall results. This would imply that although a strong signal can be detected even with a small number of traits, well-chosen variables that give a higher definition of shape will enhance the capability of the analyses to capture weaker signals. Hence, the number of variables and an accurate representation of the general shape are important factors to take into consideration in this type of study.

Other sources of variation are of course possible, such as differences in activity levels and diet, which could in principle affect pelvic shape [37], [87]–[89]. Being impossible to test for these potentially confounding variables with the present dataset, we can only assume that part of the variation in pelvic shape in our samples could be accounted for by plastic reactions to unknown factors. However, even if present, their effect is not strong enough to obliterate the signature of ancient population history.

Our analyses testing for obstetrical constraints in shape variation indicated no difference in the neutral OoA pattern between males and females, a difference that would be expected if constraints were stronger in females than in males. This result is consistent with Tague’s [51] finding that males are not necessarily more variable in pelvic morphology than females. It is also consistent with recent suggestions that the obstetric dilemma may be influenced more by maternal energetics than pelvic morphology per se [90], [91]. However, it is important to note that our analyses did not include the sacrum, which is also a component of variation in obstetric dimensions (although see [31]). Indeed, the demographic signature does appear to be weaker in certain functional (i.e. the obstetrically related) regions of the os coxae than in others. The fact that the false pelvis shows a stronger neutral pattern of variation, and a higher overall average variation in shape than the (obstetrically-related) true pelvis, suggests that obstetrical constraints might indeed have an influence on some pelvic traits. At the very least, our analyses indicate that different regions of the pelvis preserve a neutral signal to different extents in a similar vein to the human cranium [7]–[9], [13], [14], [22], [92], [93].

In summary, the shape of the os coxae reveals a surprisingly strong signature of ancient population history and an overall neutral pattern of variance apportionment, despite the fact that some aspects of pelvic form have been demonstrated to be subject to selective pressures (e.g., [32], [33], [35], [60]). Moreover, although our results reveal some potential for obstetrical effects to be exerting an effect on population variance, these are not preeminent in terms of obliterating the underlying neutral pattern. However, it is important to underline that the present results, based on *within*-population shape diversity, cannot be interpreted as an indication of no effect of selective factors on pelvic shape differences *between* populations. Indeed, our results are in line with suggestions that the effects of climatic selection in the human pelvis may have been more prevalent on aspects of size (via Bergmann’s [63] rule) than they have on global patterns of *shape* variation [31]. Future work should investigate the extent to which climatic selection has contributed to deviations from total fit to the neutral model; assessing its contribution in *relative* rather than absolute terms.

Our overall main finding of a substantial component of neutral shape variation in the human pelvis is important in the light of studies of the human cranium (e.g., [2], [6]–[9], [94]). Such studies have highlighted that human cranial variation can largely be thought of as a proxy for neutral genetic variation in terms of understanding past population history [1], [15], leading to insights into factors such as the divergence of Neanderthals and modern humans [95], [96], the dispersal of modern humans [10], [30], [97], the demographic signature of the Neolithic expansion [98], [99], and the settlement of the Americas (e.g., [100]–[102]). Our study indicates that shape data for the human pelvis might provide an important adjunct to, or substitute for, cranial data in anthropological questions of this type.

## References

[1] Roseman CC, Weaver TD (2007) Molecules versus morphology? Not for the human cranium. BioEssays 29: 1185–1188. 18008372 10.1002/bies.20678

[2] Relethford JH (1994) Craniometric variation among modern human populations. Am J Phys Anthropol 95: 53–62. 7527996 10.1002/ajpa.1330950105

[3] Relethford JH (2002) Apportionment of global human genetic diversity based on craniometrics and skin color. Am J Phys Anthropol 118: 393–398. 12124919 10.1002/ajpa.10079

[4] Relethford JH (2004b) Global patterns of isolation by distance based on genetic and morphological data. Hum Biol 76: 499–513. 15754968 10.1353/hub.2004.0060

[5] González-José R, Van der Molen S, González-Pérez E, Hernández M (2004) Patterns of phenotypic covariation and correlation in modern humans as viewed from morphological integration. Am J Phys Anthropol 123: 69–77. 14669238 10.1002/ajpa.10302

[6] Roseman CC (2004) Detecting interregionally diversifying natural selection on modern human cranial form by using matched molecular and morphometric data. Proc Natl Acad Sci U S A 101: 12824–12829. 15326305 10.1073/pnas.0402637101 PMC516480

[7] Roseman CC, Weaver TD (2004) Multivariate apportionment of global human craniometric diversity. Am J Phys Anthropol 125: 257–263. 15386236 10.1002/ajpa.10424

[8] Harvati K, Weaver TD (2006a) Human cranial anatomy and the differential preservation of population history and climate signatures. Anat Rec A 288: 1225–1233. 10.1002/ar.a.20395 17075844

[9] Harvati K, Weaver TD (2006b). Reliability of cranial morphology in reconstructing Neanderthal phylogeny. In: Harvati K, Harrison T, editors. Neanderthals revisited: New approaches and perspectives. Dordrecht: Springer Netherlands. 239–254.

[10] Manica A, Amos W, Balloux F, Hanihara T (2007) The effect of ancient population bottlenecks on human phenotypic variation. Nature 448: 346–349. 17637668 10.1038/nature05951 PMC1978547

[11] Hanihara T (2008) Morphological variation of major human populations based on nonmetric dental traits. Am J Phys Anthropol 136: 169–182. 18257017 10.1002/ajpa.20792

[12] Betti L, Balloux F, Hanihara T, Manica A (2009) Ancient demography, not climate, explains within-population phenotypic diversity in humans. Proc Royal Soc, B 276: 809–814. 10.1098/rspb.2008.1563 PMC2664379 19129123

[13] Smith HF (2009) Which cranial regions reflect molecular distances reliably in humans? Evidence from three-dimensional morphology. Am J Hum Biol 21: 36–47. 18663742 10.1002/ajhb.20805

[14] von Cramon-Taubadel N (2009a) Congruence of individual cranial bone morphology and neutral molecular affinity patterns in modern humans. Am J Phys Anthropol 140: 205–215. 19418568 10.1002/ajpa.21041

[15] von Cramon-Taubadel N, Weaver TD (2009) Insights from a quantitative genetic approach to human morphological evolution. Evol Anthropol 18: 237–240.

[16] Lewontin R (1972) The apportionment of human diversity. Evol Biol 6: 381–398.

[17] Barbujani G, Magagni A, Minch E, Cavalli-Sforza LL (1997) An apportionment of human DNA diversity. Proc Natl Acad Sci U S A 94: 4516–4519. 9114021 10.1073/pnas.94.9.4516 PMC20754

[18] Jorde LB, Watkins WS, Bamshad MJ, Dixon ME, Ricker CE, et al (2000) The distribution of human genetic diversity: A comparison of mitochondrial, autosomal, and Y-chromosome data. Am J Hum Genet 66: 979–988. 10712212 10.1086/302825 PMC1288178

[19] Long JC (2009) Update to Long and Kittles’s “Human genetic diversity and the nonexistence of biological races” (2003): Fixation on an index. Hum Biol 81: 799–803. 20504197 10.3378/027.081.0622

[20] Long JC, Kittles RA (2003) Human genetic diversity and the nonexistence of biological races. Hum Biol 75: 449–471. 14655871 10.1353/hub.2003.0058

[21] Edwards AWF (2003) Human genetic diversity: Lewontin’s fallacy. BioEssays 25: 798–801. 12879450 10.1002/bies.10315

[22] von Cramon-Taubadel N (2009b) Revisiting the homoiology hypothesis: the impact of phenotypic plasticity on the reconstruction of human population history from craniometric data. J Hum Evol 57: 179–190. 19604539 10.1016/j.jhevol.2009.05.009

[23] von Cramon-Taubadel N (2011b) The relative efficacy of functional and developmental cranial modules for reconstructing global human population history. Am J Phys Anthropol 146: 83–93. 21710659 10.1002/ajpa.21550

[24] Cavalli-Sforza LL, Menozzi P, Piazza A (1994). The history and geography of human genes. Princeton: Princeton University Press.

[25] Eller E (1999) Population substructure and isolation by distance in three continental regions. Am J Phys Anthropol 108: 147–159. 9988378 10.1002/(SICI)1096-8644(199902)108:2<147::AID-AJPA2>3.0.CO;2-E

[26] Betti L, Balloux F, Hanihara T, Manica A (2010) The relative role of drift and selection in shaping the human skull. Am J Phys Anthropol 141: 76–82. 19582777 10.1002/ajpa.21115

[27] Prugnolle F, Manica A, Balloux F (2005) Geography predicts neutral genetic diversity of human populations. Curr Biol 15: R159–160. 15753023 10.1016/j.cub.2005.02.038 PMC1800886

[28] Ramachandran S, Deshpande O, Roseman CC, Rosenberg NA, Feldman MW, et al (2005) Support from the relationship of genetic and geographic distance in human populations for a serial founder effect originating in Africa. Proc Natl Acad Sci U S A 102: 15942–15947. 16243969 10.1073/pnas.0507611102 PMC1276087

[29] Li JZ, Absher DM, Tang H, Southwick AM, Casto AM, et al (2008) Worldwide human relationships inferred from genome-wide patterns of variation. Science 319: 1100–1104. 18292342 10.1126/science.1153717

[30] von Cramon-Taubadel N, Lycett SJ (2008) Human cranial variation fits iterative founder effect model with African origin. Am J Phys Anthropol 136: 108–113. 18161847 10.1002/ajpa.20775

[31] Betti L, von Cramon-Taubadel N, Lycett SJ (2012) Human pelvis and long bones reveal differential preservation of ancient population history and migration out of Africa. Hum Biol 84: 139–152. 22708818 10.3378/027.084.0203

[32] Ruff CB (1993) Climatic adaptation and hominid evolution: The thermoregulatory imperative. Evol Anthropol 2: 53–60.

[33] Ruff CB (1994) Morphological adaptation to climate in modern and fossil hominids. Am J Phys Anthropol 37: 65–107.

[34] Holliday TW (1997a) Body proportions in Late Pleistocene Europe and modern human origins. J Hum Evol 32: 423–447. 9169992 10.1006/jhev.1996.0111

[35] Holliday TW (1997b) Postcranial evidence of cold adaptation in European Neandertals. Am J Phys Anthropol 104: 245–258. 9386830 10.1002/(SICI)1096-8644(199710)104:2<245::AID-AJPA10>3.0.CO;2-#

[36] Stock J, Pfeiffer S (2001) Linking structural variability in long bone diaphyses to habitual behaviors: Foragers from the southern African Later Stone Age and the Andaman Islands. Am J Phys Anthropol 115: 337–348. 11471132 10.1002/ajpa.1090

[37] Stock JT (2006) Hunter-gatherer postcranial robusticity relative to patterns of mobility, climatic adaptation, and selection for tissue economy. Am J Phys Anthropol 131: 194–204. 16596600 10.1002/ajpa.20398

[38] Holliday TW, Hilton CE (2010) Body proportions of circumpolar peoples as evidenced from skeletal data: Ipiutak and Tigara (Point Hope) versus Kodiak Island Inuit. Am J Phys Anthropol 142: 287–302. 19927367 10.1002/ajpa.21226

[39] Waterman HC (1929) Studies in the evolution of the pelvis of man and other primates. Bull Am Mus Nat Hist 58: 585–642.

[40] Reynolds ED (1931) The evolution of the human pelvis in relation to the mechanics in the erect posture. Pap Peabody Mus 9: 255–334.

[41] Schultz AH (1936) Characters common to higher primates and characters specific for man (continued). Q Rev Biol 11: 425–455.

[42] McHenry HM, Corruccini RS (1978) Analysis of the hominoid os coxae by Cartesian coordinates. Am J Phys Anthropol 48: 215–226. 416722 10.1002/ajpa.1330480216

[43] Steudel K (1978) A multivariate analysis of the pelvis of early hominids. J Hum Evol 7: 583–595.

[44] Berge C (1984) Multivariate analysis of the pelvis for hominids and other extant primates: Implications for the locomotion and systematics of the different species of australopithecines. J Hum Evol 13: 555–562.

[45] Lovejoy CO (1988) Evolution of human walking. Sci Am 259: 118–125. 10.1038/scientificamerican1188-118 3212438

[46] Tague RG (1992) Sexual dimorphism in the human bony pelvis, with a consideration of the Neandertal pelvis from Kebara Cave, Israel. Am J Phys Anthropol 88: 1–21. 1510108 10.1002/ajpa.1330880102

[47] Ward CV (2002) Interpreting the posture and locomotion of *Australopithecus afarensis*: Where do we stand*?* . Am J Phys Anthropol 119: 185–215. 10.1002/ajpa.10185 12653313

[48] Weaver TD, Hublin JJ (2009) Neandertal birth canal shape and the evolution of human childbirth. Proc Natl Acad Sci U S A 106: 8151–8156. 19380728 10.1073/pnas.0812554106 PMC2688883

[49] DeSilva JM (2011) A shift toward birthing relatively large infants early in human evolution. Proc Natl Acad Sci U S A 108: 1022–1027. 21199942 10.1073/pnas.1003865108 PMC3024680

[50] Thoms H, Foote WR, Friedman I (1939) The clinical significance of pelvic variations: A dimensional study of the upper, mid, and lower pelvis in 200 white primiparous women. Am J Obstet Gynecol 38: 634–642.

[51] Tague RG (1989) Variation in pelvic size between males and females. Am J Phys Anthropol 80: 59–71. 2801906 10.1002/ajpa.1330800108

[52] Rosenberg KR (1992) The evolution of modern human childbirth. Am J Phys Anthropol 35: 89–124.

[53] Ruff C (2002) Variation in human body size and shape. Annu Rev Anthropol 31: 211–232.

[54] Cox M, Scott A (1992) Evaluation of the obstetric significance of some pelvic characters in an 18th century British sample of known parity status. Am J Phys Anthropol 89: 431–440. 1463087 10.1002/ajpa.1330890404

[55] Correia H, Balseiro S, De Areia M (2005) Sexual dimorphism in the human pelvis: Testing a new hypothesis. HOMO 56: 153–160. 16130838 10.1016/j.jchb.2005.05.003

[56] Kurki HK (2011) Pelvic dimorphism in relation to body size and body size dimorphism in humans. J Hum Evol 61: 631–643. 21981862 10.1016/j.jhevol.2011.07.006

[57] Krogman WM (1951) The role of physical anthropology in dental and medical research. Am J Phys Anthropol 9: 211–218. 14868774 10.1002/ajpa.1330090208

[58] Washburn SN (1960) Tools and human evolution. Sci Am 203: 3–15. 13843002

[59] Rosenberg K, Trevathan W (1995) Bipedalism and human birth: The obstetrical dilemma revisited. Evol Anthropol 4: 161–168.

[60] Weaver TD (2002). A multi-causal functional analysis of hominid hip morphology. Palo Alto: Stanford University. Unpublished PhD Thesis.

[61] Ruff CB (1991) Climate and body shape in hominid evolution. J Hum Evol 21: 81–105.

[62] Ruff C (2010) Body size and body shape in early hominins - implications of the Gona Pelvis. J Hum Evol 58: 166–178. 19945140 10.1016/j.jhevol.2009.10.003

[63] Bergmann C (1847) Uber die verhaltniesse der warmeokonomie der thiere zu ihrer grosse. Gotteningen Studien 1: 595–708.

[64] Tague RG (1995) Variation in pelvic size between males and females in nonhuman anthropoids. Am J Phys Anthropol 97: 213–233. 7573375 10.1002/ajpa.1330970302

[65] Kurki HK (2007) Protection of obstetric dimensions in a small-bodied human sample. Am J Phys Anthropol 133: 1152–1165. 17530697 10.1002/ajpa.20636

[66] Aiello LC, Dean C (1990). An introduction to human evolutionary anatomy. London: Academic Press.

[67] Phenice TW (1969) A newly developed visual method of sexing the os pubis. Am J Phys Anthropol 30: 297–301. 5772048 10.1002/ajpa.1330300214

[68] Işcan MY, Derrick K (1984) Determination of sex from sacroiliac joint: A visual assessment technique. Florida Scientist 47: 94–98.

[69] Sutherland LD, Suchey JM (1991) Use of the ventral arc in pubic sex determination. J Forensic Sci 36: 501–511. 2066725

[70] von Cramon-Taubadel N, Frazier BC, Lahr MM (2007) The problem of assessing landmark error in geometric morphometrics: Theory, methods, and modifications Am J Phys Anthropol. 134: 24–35. 10.1002/ajpa.20616 17503448

[71] Rohlf FJ (1990) Morphometrics. Annu Rev Ecol Syst 21: 299–316.

[72] Rohlf FJ, Slice D (1990) Extensions of the Procrustes method for the optimal superimposition of landmarks. Syst Biol 39: 40–59.

[73] Rohlf FJ, Marcus LF (1993) A revolution in morphometrics. Trends Ecol Evol 8: 129–132. 21236128 10.1016/0169-5347(93)90024-J

[74] Slice DE (1996). Three-dimensional generalized resistance fitting and the comparison of least-squares and resistant fit residuals. In: Marcus LF, Corti M, Loy A, Naylor GJP, Slice DE, editors. Advances in morphometrics. New York: Plenum Press. 179–199.

[75] O’Higgins P, Jones N (1998) Facial growth in *Cercocebus torquatus*: an application of three-dimensional geometric morphometric techniques to the study of morphological variation. J Anat 193: 251–272. 9827641 10.1046/j.1469-7580.1998.19320251.x PMC1467845

[76] R Development Core Team (2011). R: A language and environment for statistical computing. Version 2.14. Vienna: R Foundation for Statistical Computing.

[77] Relethford JH, Crawford MH, Blangero J (1997) Genetic drift and gene flow in post-famine Ireland. Hum Biol 69: 443–465. 9198306

[78] Harpending H, Jenkins T (1973). Genetic distance among Southern African populations. In: Crawford MH, Workman PL, editors. Methods and theories of anthropological genetics. Albuquerque: University of New Mexico Press. 177–199.

[79] Relethford JH, Blangero J (1990) Detection of differential gene flow from patterns of quantitative variation. Hum Biol 62: 5–25. 2323770

[80] Zelditch ML, Swiderski DL, Sheets HD, Fink WL (2004). Geometric Morphometrics for Biologists: A Primer. London: Elsevier Academic Press.

[81] Franklin D, Cardini A, Oxnard CE (2010) A geometric morphometric approach to the quantification of population variation in sub-Saharan African crania. Am J Hum Biol 22: 23–35. 19309683 10.1002/ajhb.20908

[82] Manica A, Prugnolle F, Balloux F (2005) Geography is a better determinant of human genetic differentiation than ethnicity. Hum Genet 118: 366–371. 16189711 10.1007/s00439-005-0039-3 PMC1805472

[83] Akaike H (1973). Information theory and an extension of the maximum likelihood principle In: Petrov BN, Csaki F, editors. Second international Symposium on Information theory. Budapest: Academiai Kiado. 267–281.

[84] Latter BDH (1980) Genetic differences within and between populations of the major human subgroups. Am Nat 116: 220–237.

[85] Ryman N, Chakraborty R, Nei M (1983) Differences in the relative distribution of human gene diversity between electrophoretic and red and white cell antigen loci. Hum Hered 33: 93–102. 6862458 10.1159/000153357

[86] Jablonski NG, Chaplin G (2000) The evolution of human skin coloration. J Hum Evol 39: 57–106. 10896812 10.1006/jhev.2000.0403

[87] Currey J (1984) Comparative mechanical properties and histology of bone. Am Zool 24: 5–12.

[88] Lieberman DE (1996) How and why humans grow thin skulls: Experimental evidence for systemic cortical robusticity. Am J Phys Anthropol 101: 217–236. 8893086 10.1002/(SICI)1096-8644(199610)101:2<217::AID-AJPA7>3.0.CO;2-Z

[89] Walker PL (2005) Greater sciatic notch morphology: Sex, age, and population differences. Am J Phys Anthropol 127: 385–391. 15693026 10.1002/ajpa.10422

[90] Dunsworth HM, Warrener AG, Deacon T, Ellison PT, Pontzer H (2012) Metabolic hypothesis for human altriciality. Proc Natl Acad Sci U S A 109: 15212–15216. 22932870 10.1073/pnas.1205282109 PMC3458333

[91] Wells JCK, DeSilva JM, Stock JT (2012) The obstetric dilemma: an ancient game of Russian roulette, or a variable dilemma sensitive to ecology? Yearb Phys Anthropol 55: 40–71. 10.1002/ajpa.22160 23138755

[92] Smith HF (2011) The role of genetic drift in shaping modern human cranial evolution: A test using microevolutionary modeling. Int J Evol Biol 2011: 145262. 21461369 10.4061/2011/145262 PMC3065169

[93] von Cramon-Taubadel N (2011a) Global human mandibular variation reflects differences in agricultural and hunter-gatherer subsistence strategies. Proc Natl Acad Sci U S A 108: 19546–19551. 22106280 10.1073/pnas.1113050108 PMC3241821

[94] Relethford JH (2004a) Boas and beyond: Migration and craniometric variation. Am J Hum Biol 16: 379–386. 15214056 10.1002/ajhb.20045

[95] Weaver TD, Roseman CC, Stringer CB (2007) Were Neandertal and modern human cranial differences produced by natural selection or genetic drift? J Hum Evol 53: 135–145. 17512036 10.1016/j.jhevol.2007.03.001

[96] Weaver TD, Roseman CC, Stringer CB (2008) Close correspondence between quantitative- and molecular-genetic divergence times for Neandertals and modern humans. Proc Natl Acad Sci U S A 105: 4645–4649. 18347337 10.1073/pnas.0709079105 PMC2290803

[97] Relethford JH (2010) Population-specific deviations of global human craniometric variation from a neutral model. Am J Phys Anthropol 142: 105–111. 19927369 10.1002/ajpa.21207

[98] Pinhasi R, von Cramon-Taubadel N (2009) Craniometric data supports demic diffusion model for the spread of agriculture into Europe. PLoS ONE 4: e6747. 19707595 10.1371/journal.pone.0006747 PMC2727056

[99] von Cramon-Taubadel N, Pinhasi R (2011) Craniometric data support a mosaic model of demic and cultural Neolithic diffusion to outlying regions of Europe. Proc Royal Soc, B 278: 2874–2880. 10.1098/rspb.2010.2678 PMC3151708 21345869

[100] Hubbe M, Harvati K, Neves W (2011) Paleoamerican morphology in the context of European and East Asian late Pleistocene variation: Implications for human dispersion into the new world. Am J Phys Anthropol 144: 442–453. 21302270 10.1002/ajpa.21425

[101] Hubbe M, Neves WA, Harvati K (2010) Testing evolutionary and dispersion scenarios for the settlement of the New World. PLoS ONE 5: e11105. 20559441 10.1371/journal.pone.0011105 PMC2885431

[102] de Azevedo S, Nocera A, Paschetta C, Castillo L, González M, et al (2011) Evaluating microevolutionary models for the early settlement of the New World: The importance of recurrent gene flow with Asia. Am J Phys Anthropol 146: 539–552. 21805463 10.1002/ajpa.21564

[103] Devor EJ (1987) Transmission of human craniofacial dimensions. J Craniofac Genet Dev Biol 7: 95–106. 3624421

[104] Williams-Blangero S, Blangero J (1992) Quantitative genetic analysis of skin reflectance: a multivariate approach. Hum Biol 64: 35–49. 1582647

